# A Matricryptic Conformation of the Integrin-Binding Domain of Fibronectin Regulates Platelet-Derived Growth Factor-Induced Intracellular Calcium Release

**DOI:** 10.3390/cells8111351

**Published:** 2019-10-30

**Authors:** Christopher S. Farrar, Geoffrey T. Rouin, Benjamin L. Miller, Carol H. Raeman, Nancie A. Mooney, Denise C. Hocking

**Affiliations:** 1 Department of Biomedical Engineering, University of Rochester, Rochester, NY 14627, USA; csfarrar89@gmail.com (C.S.F.); geoffrey.rouin@westpharma.com (G.T.R.); benjamin_miller@urmc.rochester.edu (B.L.M.); carol.raeman@rochester.edu (C.H.R.); nmooney@stanford.edu (N.A.M.); 2 Department of Dermatology, University of Rochester School of Medicine and Dentistry, Rochester, NY 14642, USA; 3 Department of Pharmacology and Physiology, University of Rochester School of Medicine and Dentistry, Rochester, NY 14642, USA

**Keywords:** extracellular matrix, calcium, phosphoinositide 3-kinase, growth factors, integrin, molecular dynamics simulations

## Abstract

Platelet-derived growth factor (PDGF) signaling is dysregulated in a wide variety of diseases, making PDGF an attractive therapeutic target. However, PDGF also affects numerous signaling cascades essential for tissue homeostasis, limiting the development of PDGF-based therapies that lack adverse side-effects. Recent studies showed that fibroblast-mediated assembly of extracellular matrix (ECM) fibronectin fibrils attenuates PDGF-induced intracellular calcium release by selectively inhibiting phosphoinositol 3-kinase (PI3K) activation while leaving other PDGF-mediated signaling cascades intact. In the present study, a series of recombinant fibronectin-derived fusion proteins were used to localize the sequences in fibronectin that are responsible for this inhibition. Results demonstrate that attenuation of PDGF-induced intracellular calcium release by the fibronectin matrix mimetic, FNIII1H,8-10 requires α5β1 integrin ligation, but is not dependent upon the matricryptic, heparin-binding site of FNIII1. Intact cell-binding fibronectin fragments were also unable to attenuate PDGF-induced intracellular calcium release. In contrast, a novel integrin-binding fragment that adopts an extended and aligned conformational state, inhibited both PI3K activation and intracellular calcium release in response to PDGF. Taken together, these studies provide evidence that attenuation of PDGF-induced intracellular calcium release by fibronectin is mediated by a novel conformation of the α5β1 integrin-binding, FNIII9-10 modules, that is expressed by fibrillar fibronectin.

## 1. Introduction

Platelet-derived growth factor (PDGF) evokes a diverse array of intracellular signaling cascades that promote a variety of cell and tissue behaviors, including cell growth [1], survival [2], chemotaxis [3], extracellular matrix (ECM) deposition [4], collagen matrix contraction [4,5] and wound healing [6]. Currently, recombinant PDGF is used for the clinical treatment of diabetic ulcers [7] and broad spectrum inhibitors of PDGF signaling are under development to treat pulmonary arterial hypertension [8] and pulmonary fibrosis [9]. However, a difficulty in targeting aberrant PDGF activity therapeutically arises from the complexity of the cellular response to PDGF, wherein broadly up- or down-regulating PDGF signaling often has undesirable consequences [1]. We recently demonstrated that the ECM form of fibronectin attenuates PDGF-induced intracellular calcium release by selectively inhibiting phosphoinositol 3-kinase (PI3K) activation while leaving other PDGF-mediated signaling cascades intact [10]. The PDGF–phospholipase C-gamma 1 (PLC-γ1)–PI3K–calcium signaling axis controls cell differentiation, proliferation, migration, and changes in gene transcription [1]. Thus, understanding the mechanism by which ECM fibronectin selectively regulates this arm of PDGF signaling has the potential to inform the design of advanced targeted therapeutics.

Fibronectin is an extensible, modular glycoprotein that binds to and influences the behavior of cells, other ECM components, and growth factors [11]. Fibronectin is secreted by cells in a soluble, protomeric form and assembled into insoluble, elastic ECM fibronectin fibrils by a cell-mediated process termed fibronectin matrix assembly [11]. Binding of the amino-terminus of soluble fibronectin to the cell surface initiates a series of conformational changes within the fibronectin protomer [12] that uncoils the central cell-binding region of fibronectin to allow for integrin engagement [13,14]. Following integrin binding, cell- and tissue-derived mechanical forces drive fibronectin fibril extension [15,16], opening homophilic binding sites for lateral and longitudinal fibril growth [17], and revealing matricryptic epitopes that further impact cell and tissue behaviors [18,19]. 

Cell adhesion to fibronectin is mediated primarily by binding interactions between α5β1 integrins and the Arg-Gly-Asp (RGD) sequence within the tenth type-III repeat of fibronectin (FNIII10) [20]. The flexible RGD loop of fibronectin is recognized by several different integrin heterodimers, including α5β1 and αvβ3 [21]. Additional amino-acid sequences in the cell-binding domain of fibronectin, including the Pro-His-Ser-Arg-Asn (PHSRN) sequence of FNIII9 and the Val-Lys-Asn-Glu-Glu-Asp (VKNEED) sequence of FNIII8, promote α5β1 integrin specificity [22,23,24,25,26]. The RGD loop of FNIII10, the PHSRN sequence of FNIII9, and the VKNEED sequence of FNIII8 are located on the same face of fibronectin, and therefore can interact simultaneously with α5β1 integrins [24,27], providing additional evidence that synergistic interactions between α5β1 integrins and RGD, PHSRN, and VKNEED sites contribute to binding affinity [28]. 

Integrin binding specificity is also affected by the conformation of the central cell-binding region of fibronectin [13,22]. Fibronectin-α5β1 interactions are dependent upon both the distance and the tilt angle between the PHRSN sequence of FNIII9 and the RGD loop of FNIII10 [25,29]. It has been proposed that cell-evolved tension can extend the type-III repeats of fibronectin and increase the distance between the PHSRN and RGD sequences from 32Å to 55Å [14]. As the distance between these sites increases, αvβ3 integrin binding remains supported, whereas α5β1 integrin ligation is attenuated [30]. Downstream consequences of integrin “switching” include reduced responsiveness of fibroblasts to vascular endothelial growth factor (VEGF) [31], enhanced angiogenesis [32], and increased VEGF secretion by preadipocytes [33]. Changes in the conformation of the cell-binding domain can also be induced directly by specific bacterial proteins that bind to the amino-terminus of fibronectin, triggering long-range conformational changes in the cell-binding domain of fibronectin that promote fibronectin-α5β1 integrin ligation [34]. 

A recombinant, fibronectin-derived fusion protein, FNIII1H,8-10, was developed previously to mimic the conformation and biological effects of the ECM form of fibronectin [18]. FNIII1H,8-10 is composed of a fragment of FNIII1 (FNIII1H), truncated at the N-terminus to expose the cryptic, heparin-binding core, that is coupled directly to the cell-binding, FNIII8-10 modules [18]. FNIII1H,8-10 binds to α5β1 integrins [35] and mimics the ability of ECM fibronectin to attenuate PDGF-induced intracellular calcium release [10]. In the present study, we used fibronectin-null mouse embryonic fibroblasts (FN-null MEFs), together with a series of recombinant FNIII1H,8-10 derivatives that selectively bind α5β1 or αvβ3 integrins, to determine the mechanism by which FNIII1H,8-10 inhibits the PDGF-PI3K-calcium signaling axis.

## 2. Materials and Methods

### 2.1. Reagents

Fibronectin was purified from outdated human plasma (American Red Cross, Rochester, NY) using gelatin-Sepharose (GE Healthcare, New York, NY, USA) affinity chromatography [36]. Collagen I was extracted from rat tail tendons, as described previously [35]. PDGF-BB was purchased from Thermo Fisher Scientific (Waltham, MA, USA). Antibodies and their sources are as follows (catalog or clone number in parentheses): 9D2 monoclonal antibody (mAb) [37] was a gift from Dr. Deane Mosher (University of Wisconsin, Madison, WI, USA); mouse IgG (I5381) and anti-vinculin mAb (V4505) antibodies were from Sigma-Aldrich; anti-phospho-PI3K pAb (4228) was from Cell Signaling Technology (Danvers, MA, USA); anti-β1 integrin (Ha2/5), anti-β3 integrin (2C9.G2), IgG (553961) and IgM (553957) isotype controls were from BD Biosciences (Mississauga, ON, Canada); and horseradish peroxidase (HRPO)-conjugated goat anti-mouse and goat anti-rabbit secondary antibodies were from BioRad (Hercules, CA, USA).

### 2.2. Cell Culture 

FN-null MEFs were cultured routinely under serum- and fibronectin-free conditions on collagen I-coated tissue culture flasks in a 1:1 mixture of Aim V (Invitrogen, Carlsbad, CA, USA) and SF Medium (Corning, Lowell, MA, USA). Fibronectin-null MEFs do not produce endogenous fibronectin but are able to polymerize exogenously-added fibronectin into a fibrillar ECM [38]. Thus, this model allows for precise control over the presence and quantity of fibronectin proteins in the culture system. Tissue culture plates were coated with type-I collagen (0.05 mg/mL in 0.02 N acetic acid) overnight at 4 °C and washed with phosphate-buffered saline (PBS) before use.

### 2.3. Recombinant Proteins 

**Figure 1:**
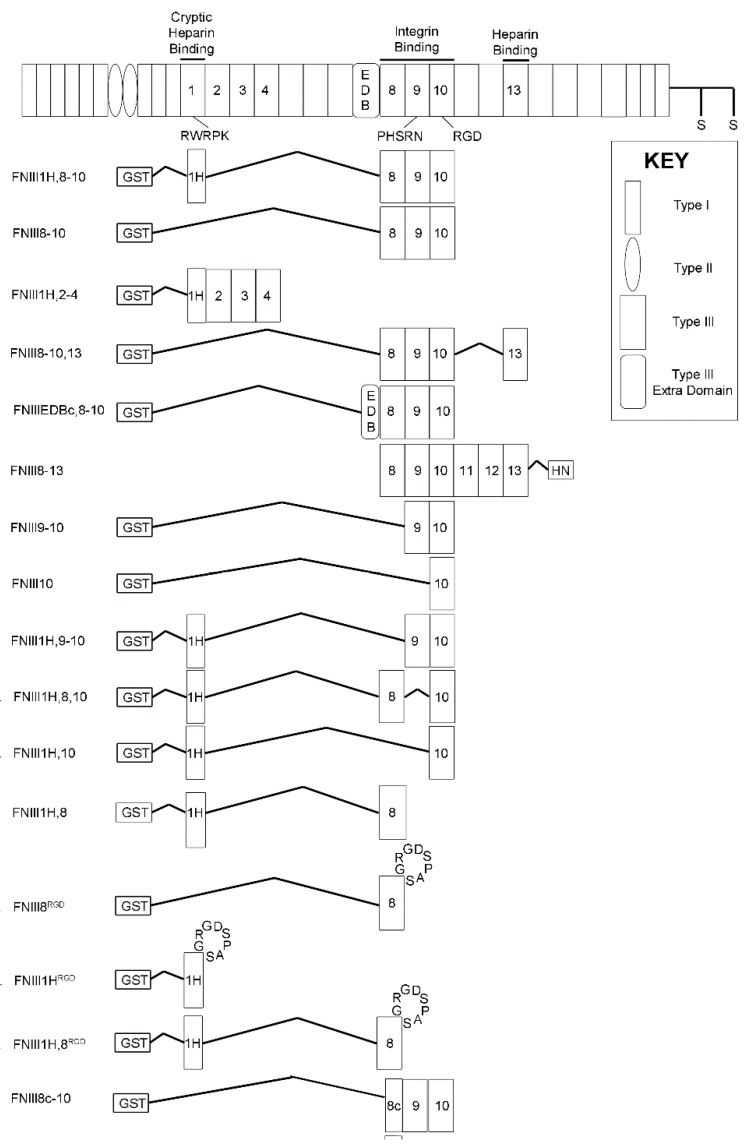
Schematic representation of a fibronectin subunit and fibronectin fusion proteins.

Construction, purification, and verification of recombinant fusion proteins have been described [18,39,40,41,42,43]. Proteins utilized in this study are illustrated schematically in Figure 1. Briefly, effects on PDGF-induced intracellular calcium release were tested using soluble, recombinant fibronectin fragments that were derived from FNIII1H,8-10 [18]. Fibronectin proteins employed include: a construct in which the cell-binding FNIII8-10 region was replaced with modules FNIII2-4 that do not bind to integrins (FNII1H,2-4); integrin-binding constructs that lacked FNIII1H (FNIII8-10, FNIII9-10, FNIII10); a construct in which the bioactive heparin-binding sequence of FNIII1H was mutated to non-charged amino acids (FNIII1H,8-10ΔRWRK); constructs in which FNIII1H was replaced with the carboxyl-terminal heparin-binding module FNIII13 (FNIII8-10,13) or a similarly truncated fibronectin extra domain-B (EDB) module (FNIIIEDBc,8-10); a series of constructs that contained FNIII1H fused to various combinations of modules from the cell-binding domain (FNIII1H,9-10; FNIII1H,8,10; FNIII1H,10; FNIII1H,8); a series of αvβ3 integrin-binding chimeric fibronectin constructs in which the integrin-binding RGD loop of FNIII10 was inserted into the analogous site of either FNIII8 or FNIII1H (FNIII8^RGD^; FNIII1H^RGD^; FNIII1H,8^RGD^); the fibronectin matrix mimetic in which the PHSRN synergy site in FNIII9 was mutated (R1374A, R1379A [26]; FNIII1H,8-10ΔPHSRN); or the fibronectin matrix mimetic with the RGD integrin-binding sequence of FNIII10 mutated to Arg-Gly-Glu (RGE) (FNIII1H,8-10ΔRGD).

Glutathione S-transferase (GST)-tagged fusion proteins were produced in *Escherichia coli* and isolated by affinity chromatography, as described previously: FNIII10 [39]; FNIII9-10 [40]; FNIII1H,8-10, FNIII8-10, FNIII1H,2-4, and FNIII8-10,13 [18]; FNIII1H,8-10ΔRGD and FNIII1H,8-10ΔPHSRN [41]; FNIII1H,8^RGD^, FNIII1H,8,10, FNIII1H,9-10, FNIII1H,10, FNIII1H,8-10ΔRRK, FNIII1H,8-10ΔRWRK, FNIIIEDBc,8-10, FNIII8^RGD^ and FNIII1H,8 [42]; FNIII1H^RGD^ [43]. For some studies, the GST tag was removed from GST/FNIII10 by digestion with 0.5 U/mg trypsin-acrylic beads (N-tosyl-L-phenylalanine chloromethyl ketone (TPCK)-treated; Thermo Fisher Scientific) for 30 min at 20 °C, and re-incubation with glutathione-Sepharose.

A fibronectin fragment spanning FNIII8 through FNIII13 (III8-13-HN; bases 3716-5623; Ala^1326^-Thr^1961^) was produced using the sense primer: 5′-A**CCATGG**CTGTTCCTCCTCCCACTGACCTGCG-3′ (NcoI site in bold) and antisense primer: 5′-G**GCGGCCGC**CAGTGGAGGCGTCGATGACCACAGG-3′ (NotI site in bold). The human fibronectin cDNA pCEF103 construct was used as a template. PCR-amplified DNA was cloned into the pEcoli-Cterm 6xHN plasmid (Clontech, Mountain View, CA), expressed in *E. coli* strain BL21(DE3), and isolated on nickel-NTA-agarose (Qiagen, Hilden, Germany). 

A fibronectin fragment comprised of a carboxyl-terminal fragment of FNIII8 with intact III9 and III10 modules (III8c-10; bases 3773-4540; Val^1305^-Thr^1600^) was produced. PCR was used to amplify human fibronectin cDNA, with pGex2T/FNIII8-10 serving as a template [18]. The sense primer for III8c-10 (5′-CCC**GGTACC**GTCACCTGGGCTCCACC-3′) contains a KpnI site (shown in bold) at the 5′ end. The antisense primer for III8c-10 (5′-CCC**CCCGGG**CTATGTTCGGTAATTATTGGAAATTGGCTTGCTG-3′) contains an Xma1 site (shown in bold) at the 5′ end. PCR-amplified DNA was cloned into pGex2T (GE Healthcare) and transfected into DH5α bacteria [18]. Proteins were isolated on gluthathione-Sepharose (GE Healthcare) and eluted with 20 mM glutathione and 2% n-octyl-β-D-glucopyranoside in PBS [44]. For all recombinant fibronectin proteins, DNA was sequenced to confirm the appropriate product. Proteins were dialyzed extensively against PBS, filter-sterilized, and purity was assessed by sodium dodecyl sulfate-polyacrylamide gel electrophoresis (SDS-PAGE) [18]. Proteins were stored in aliquots at −80 °C.

### 2.4. Intracellular Calcium Release 

FN-null MEFs were seeded at 1.1 × 10^5^ cells/cm^2^ in Dulbecco’s modified Eagle’s medium (DMEM) (Gibco #11965-092) onto black, clear-bottom, 96-well plates precoated with collagen. Four hours after seeding, fibronectin or fusion proteins were added to the media. Following an overnight incubation (~23 h post-seeding), cells were well-spread and confluent. Media was removed and cells were loaded with 4.6 μM Fluo-4 AM (Molecular Probes; Eugene, OR, USA) in a 4-(2-hydroxyethyl)-1-piperazineethanesulfonic acid (HEPES)-based imaging buffer (127 nM NaCl, 0.56 mM MgCl_2_, 4.7 mM KCl, 0.55 mM Na_2_HPO_4_, 1.28 mM CaCl_2_, 10 mM HEPES-NaOH, 11 mM D-glucose, pH 7.4) [45]. After 40 min, the Fluo-4-containing imaging buffer was replaced with calcium-free imaging buffer containing 200 μM ethylene glycol tetraacetic acid (EGTA), and cells were incubated for 20 min to allow for intracellular de-esterification of Fluo-4. Fluorescence intensity was measured using a FlexStation Multi-Mode Microplate Reader (Molecular Devices, San Jose, CA, USA). This device allows for concurrent scanning of all 8 wells within a single column of the 96-well plate, taking a fluorescence reading for each of the 8 wells every 1.5 s. Baseline fluorescence intensity measurements were recorded for 30 s prior to the addition of PDGF-BB. Control wells received an equal volume of vehicle (PBS with 0.1% bovine serum albumin (BSA)).

To quantify changes in intracellular calcium in response to PDGF, raw data were filtered to reduce noise inherent in the fluorescence signal by taking a 30 s running average of the raw data [46]. The PDGF-induced fluorescence increase was defined as the peak increase in fluorescence intensity (ΔF) above baseline fluorescence (F_0_). Data are presented as the relative change in fluorescence (ΔF/F_0_) [46]. Data are expressed as mean ± the standard error of the mean (SEM) of ΔF/F_0_ from at least 3 experiments performed in at least triplicate. ΔF/F_0_ values from individual trials were normalized to a control condition (e.g., “+GST”), which was set to 1.

### 2.5. Immunoblotting 

Cells were solubilized in Laemmli buffer and lysates (~40 μg protein per lane) were analyzed by SDS-PAGE and immunoblotting, as described previously [44]. Immunoblots were blocked with 3% BSA (Heat-Shock Fraction V, pH = 7.5; Affymetrix, Cleveland, OH, USA) in Tris-buffered saline with Tween 20 (TBS-T) (20 mM Tris-HCl pH 7.6, 137 mM NaCl, 1% Tween 20) and incubated with anti-vinculin mAb (1:2000) or anti-phospho-PI3K pAb (1:2000) overnight at 4 °C. Blots were washed with TBS-T, incubated with goat anti-rabbit (1:5000) or goat anti-mouse (1:20,000) horseradish peroxidase-linked secondary antibodies, and developed using SuperSignal^®^ West Pico Chemiluminescent Substrate (Thermo Fisher Scientific). The average net band intensity for duplicate samples was determined by densitometry using Carestream Molecular Imaging Software (Rochester, NY, USA). Relative band densities were averaged across multiple experiments.

### 2.6. Molecular Dynamics Simulations 

All calculations were conducted using version 9.1.107 of Maestro (Schrödinger, LLC, New York, NY, USA) with the Amber* force field. The crystallographic structure of FNIII8-10 was obtained from FNIII7-10 [27] (Protein Data Bank code 1FNF). The total energy in the Amber* force field includes both bond energies (e.g., bond stretching, angle bending, and torsional energies) and non-bonded interactions (e.g., van der Walls and electrostatic interactions) between the atoms in a molecule. Simulations utilized the generalized Born and solvent accessible surface area (GB/SA) continuum model [47], water as a solvent, and a uniform dielectric constant of 1.0. Maestro was set to compute stochastic dynamics, with “shake” on bonds to hydrogens for all-atom simulations. The computations were performed assuming a temperature of 27 °C, with a 1.5 femtosecond timestep for 10,000 picoseconds. The equilibration time was 1.0 picosecond. FNIII8-10 was simulated with a united atom model, while FNIII8c-10 was simulated using both all-atom and united atom models. At least 2 replicas of 10 ns were performed for each protein. All distances were measured using Cα carbons. The distance between the RGD and synergy site was defined as the distance between R^1379^ in FNIII9 and R^1493^ in FNIII10. The rotational angle was defined as the dihedral angle formed by the Cα carbons of R^1379^ in FNIII9 and R^1493^ in FNIII10 and the centers of mass of FNIII9 (S^1396^) and FNIII10 (T^1486^) [14].

### 2.7. Cell Attachment Assays

FN-null MEFs were preincubated in suspension with anti-β1 or -β3 integrin blocking antibodies (25 μg/mL), immunoglobulin G or M (IgG or IgM) isotype controls (25 μg/mL), or 10 mM ethylenediaminetetraacetic acid (EDTA), for 40 min at 37 °C. Cells were seeded (9.4 × 10^4^ cells/cm^2^) in 96-well plates, pre-coated with saturating concentrations (125 nM) of FNIII8-10, FNIII9-10, or FNIII10, and incubated for 15 min at 4 °C. For competition assays, cells were pre-incubated with various concentrations of FNIII8-10 or FNIII8c-10 for 15 min prior to seeding onto FNIII8-10-coated wells. Cell attachment was determined by staining with 0.1% crystal violet, as described previously [42]. 

### 2.8. Statistical Analyses 

Data are presented as mean fold increase ± the standard error of the mean (SEM) relative to control treatment. Experiments were performed in quadruplicate (calcium signaling, cell adhesion) or duplicate (immunoblotting) for a minimum of 3 independent experiments. Statistical analysis was performed using Prism, Version 4 (GraphPad, LaJolla, CA, USA). Unless otherwise indicated, statistical significance was determined using an analysis of variance (ANOVA) with a Bonferroni’s post-test. Results were considered significant if *P* values were less than 0.05.

## 3. Results

### 3.1. Effects of Cell-Binding Fibronectin Fragments on Platelet-Derived Growth Factor (PDGF)-Induced Intracellular Calcium Release 

**Figure 2:**
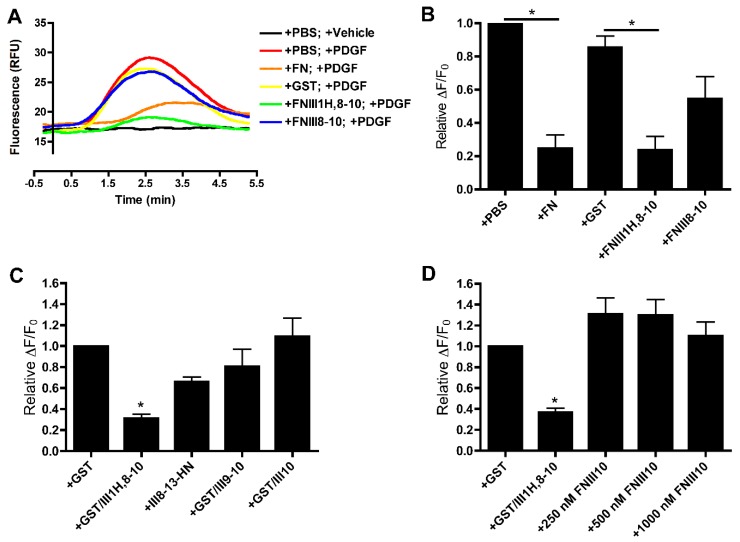
Integrin ligation with cell-binding fibronectin fragments does not attenuate platelet-derived growth factor (PDGF)-induced intracellular calcium release. Fibronectin-null mouse embryonic fibroblasts (FN-null MEFs) were seeded on collagen-coated wells (1.1 × 10^5^ cells/cm^2^). Four hours after seeding, cells were treated with fibronectin (FN; 25 nM) or an equal volume of phosphate-buffered saline (PBS); the indicated fibronectin fusion protein (250 nM); or the indicated concentrations of tag-less FNIII10. After 20 h, cells were loaded with Fluo-4, stimulated with 30 ng/mL PDGF and monitored for changes in fluorescence intensity. (**A**) Each trace represents fluorescence intensity versus time for a population of cells in an individual well. Data are representative of 1 of 4 independent experiments performed in quadruplicate. (**B**–**D**) Data are presented as relative change in fluorescence intensity from baseline (ΔF/F_0_) and are expressed as mean ± the standard error of the mean (SEM) of 4 (**B**) or 7 (**C**,**D**) experiments performed in quadruplicate. Values were normalized to “+PBS” or “+glutathione S-transferase (GST)”, which were set to 1. (**B**) * Significantly different versus respective control by analysis of variance (ANOVA) (*p* < 0.05). (**C**,**D**) * Significantly different versus +GST by ANOVA (*p* < 0.05).

Fibronectin matrix assembly by either FN-null MEFs or human dermal fibroblasts reduces the magnitude of intracellular calcium release in response to PDGF exposure [10]. A similar inhibition of PDGF-induced calcium release was observed following treatment of FN-null MEFs with the recombinant fibronectin matrix mimetic, FNIII1H,8-10 [10]. In the present study, a series of fibronectin fragments was used in conjunction with FN-null MEFs to localize the sites in FNIII1H,8-10 responsible for this inhibitory activity. To determine if integrin ligation alone attenuates PDGF-induced intracellular calcium release, collagen-adherent FN-null MEFs were pretreated with various fibronectin fragments spanning the integrin-binding domain, and then stimulated with PDGF. Fluorescence intensity was recorded as an indicator of intracellular calcium concentration. As demonstrated previously [10,48], PDGF evoked a single, transient increase in intracellular calcium that peaked ~2.5 min after PDGF addition (Figure 2A, +PBS; +PDGF). No significant change in fluorescence intensity was observed after the addition of vehicle only (Figure 2A, +PBS; +Vehicle). As reported [10], cells pre-exposed to either fibronectin or FNIII1H,8-10 exhibited an attenuated response to PDGF compared to the respective controls (Figure 2A,B: +FN versus +PBS; +FNIII1H,8-10 versus +GST). In contrast, pre-exposing cells to FNIII8-10 did not alter PDGF-induced intracellular calcium release (Figure 2A,B). Similarly, pretreating cells with either longer (FNIII8-13) or shorter integrin-binding fibronectin fragments (FNIII9-10 and FNIII10) did not affect PDGF-mediated intracellular calcium release (Figure 2C). PDGF-induced intracellular calcium release was also not inhibited with tag-less FNIII10 fragments at concentrations up to 1 μm (Figure 2D). Taken together, these data indicate that integrin ligation with intact cell-binding fibronectin fragments is not sufficient to inhibit intracellular calcium release in response to PDGF.

### 3.2. Role of FNIII1H in Attenuating PDGF-Induced Intracellular Calcium Release 

**Figure 3:**
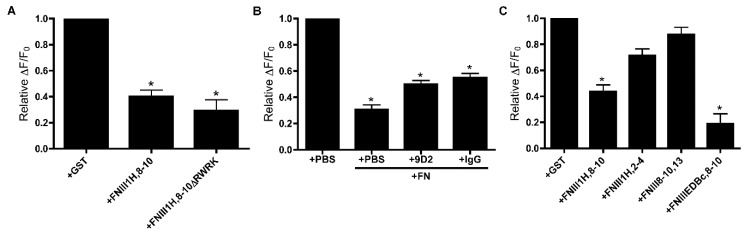
The matricryptic FNIII1H site is not essential for fibronectin-mediated attenuation of PDGF-induced intracellular calcium release. FN-null MEFs were seeded on collagen-coated wells (1.1 × 10^5^ cells/cm^2^). Four hours after seeding, cells were treated for 20 h with (**A**,**C**) the indicated fibronectin (FN) fusion proteins (250 nM) or (**B**) FN (25 nM). Cells were loaded with Fluo-4, stimulated with 30 ng/mL PDGF, and monitored for changes in fluorescence intensity. In (**B**), cells were treated with 9D2 mAb (25 μg/mL) or non-immune IgG (25 μg/mL) or an equal volume of PBS, 20 min prior to PDGF treatment. Data are presented as relative change in fluorescence intensity from baseline (ΔF/F_0_) and are expressed as mean ± SEM of 3 (**A**,**C**) or 6 (**B**) experiments performed in quadruplicate. Values were normalized to the “+GST” condition (**A**,**C**) or the “+PBS” condition (**B**), which was set to 1. * Significantly different versus +GST (**A**,**C**) or +PBS (**B**) by ANOVA (*p* < 0.05).

FNIII1 contains a matricryptic, heparin binding sequence, Arg^613^-Trp^614^-Arg^615^-Pro^616^-Lys^617^ (RWRPK) [41,49]. Mutating this sequence in FNIII1H,8-10 abolishes heparin-binding activity [41] and the ability of FNIII1H,8-10 to stimulate cell proliferation and arteriolar vasodilation [41,49]. To determine whether the RWRPK sequence is necessary for attenuation of PDGF-induced intracellular calcium release by FNIII1H,8-10, FN-null MEFs were cultured overnight in the presence of GST, FNIII1H,8-10, or FNIII1H,8-10ΔRWRK, and PDGF-induced intracellular calcium release was measured. Both FNIII1H,8-10 and FNIII1H,8-10ΔRWRK caused a similar, significant reduction in PDGF-induced intracellular calcium release compared to treatment with the GST control (Figure 3A), indicating that the RWRPK sequence of FNIII1H is not necessary for FNIII1H,8-10-mediated attenuation of PDGF-induced calcium release.

The anti-fibronectin monoclonal antibody, 9D2, binds to and blocks the biological activity of the RWRPK site of FNIII1 in full-length fibronectin [19,50]. Thus, to further examine a role for the matricryptic site of FNIII1 in regulating PDGF-induced calcium release, we asked if blocking the matricryptic site in cell-assembled fibronectin fibrils could restore the calcium response to PDGF. FN-null MEFs were supplied with exogenous fibronectin and allowed to polymerize a fibronectin matrix overnight, then treated with either 9D2 or non-immune IgG prior to PDGF addition. As shown in Figure 3B, the addition of 9D2 mAbs did not abolish the inhibitory effect of fibronectin on PDGF-induced intracellular calcium release, and effects were not different than IgG-treated controls. These results provide additional evidence that the bioactive RWRPK sequence of FNIII1H does not play a role in fibronectin-mediated attenuation of PDGF-induced intracellular calcium release.

Thus far, the results indicate that neither the matricryptic site of FNIII1 nor integrin-binding fragments inhibit calcium release in response to PDGF. Therefore, to further explore the mechanism of FNIII1H,8-10 inhibition, we analyzed a series of FNIII1H,8-10 variants for changes in activity. Replacing FNIII8-10 with the non-integrin-binding modules, FNIII2-4 (FNIII1H,2-4), resulted in a loss of activity (Figure 3C), pointing back to a role for the integrin-binding domain in the inhibition of calcium release. However, replacing FNIII1H with a heparin-binding domain at the C-terminus of FNIII8-10 (FNIII8-10,13) also resulted in loss of activity (Figure 3C), suggesting that amino acid sequences N-terminal to FNIII8-10 may be required for activity. Indeed, a construct in which FNIII1H was replaced with another truncated FNIII fragment placed N-terminal to FNIII8-10 (FNIIIEDBc,8-10) significantly attenuated PDGF-induced intracellular calcium release compared to the GST control (Figure 3C). Moreover, the extent of inhibition by FNIIIEDBc,8-10 was indistinguishable from that of FNIII1H,8-10 (Figure 3C). Together, these results suggest that coupling a truncated FNIII module to the amino-terminus of FNII8-10 confers the biological activity of inhibiting PDGF-induced intracellular calcium release.

### 3.3. Role of FNIII9 and FNIII10 in Attenuating PDGF-Induced Intracellular Calcium Release

**Figure 4:**
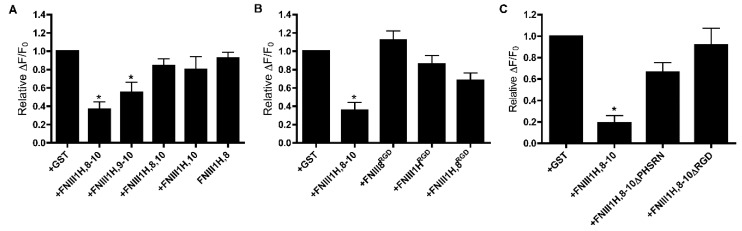
FNIII9 and FNIII10 are necessary for attenuation of PDGF-induced intracellular calcium release. FN-null MEFs were seeded on collagen-coated wells (1.1 × 10^5^ cells/cm^2^). Four hours after seeding, cells were treated with the indicated fibronectin fusion proteins (250 nM) and incubated for 20 h. Cells were loaded with Fluo-4, stimulated with 30 ng/mL PDGF, and monitored for changes in fluorescence intensity. Data are presented as relative change in fluorescence intensity from baseline (ΔF/F_0_) and are expressed as mean ± SEM of 8 (**A**,**B**) or 5 (**C**) experiments performed in quadruplicate. Values were normalized to the “+GST” condition, which was set to 1. * Significantly different versus +GST by ANOVA (*p* < 0.05).

FNIII8-10 contains three FNIII domains that work together to promote α5β1 integrin ligation [24,27]. FNIII1H,8-10 ligates α5β1 integrins, while removing individual FNIII modules from the cell-binding domain of this construct shifts integrin specificity towards αvβ3 integrins [42]. Thus, to ask directly which modules of the cell-binding domain of fibronectin mediate the effect of FNIII1H,8-10 on PDGF-calcium signaling, we analyzed PDGF-induced intracellular calcium release in response to integrin-binding variants of FNIII-1H,8-10. A construct in which FNIII8 was removed (FNIII1H,9-10) attenuated PDGF-induced intracellular calcium release to a similar extent as FNIII1H,8-10 (Figure 4A), indicating that FNIII8 is not necessary for activity. In contrast, constructs in which FNIII9 (FNIII1H,8,10; FNIII1H,10) and FNIII10 (FNIII1H,8) were removed no longer inhibited PDGF-induced intracellular calcium release (Figure 4A), indicating a role for FNIII9-10. Inserting the integrin-binding RGD loop into the analogous site in either FNIII1H (FNIII1H^RGD^) or FNIII8 (FNII8^RGD^; FNIII1H,8^RGD^) creates constructs that selectively ligate αvβ3 integrins [35,42]. None of these chimeric constructs inhibited PDGF-induced intracellular calcium release (Figure 4B), providing additional evidence that attenuation of PDGF-induced intracellular calcium release is mediated by α5β1 integrins, and not by αvβ3 integrins.

Binding of α5β1 integrins to FNIII9-10 requires the RGD loop of FNIII10 and the PHSRN “synergy sequence” of the neighboring FNIII9 module [22,25,26,51]. Therefore, FNIII1H,8-10 constructs that contain mutations in either the PHSRN (FNIII1H,8-10ΔPHSRN) or RGD (FNIII1H,8-10ΔRGD) sequences were next used to assess the role of α5β1 integrin ligation in FNIII1H,8-10-mediated attenuation of calcium release. As shown in Figure 4C, intracellular calcium release was attenuated in cells pretreated with FNIII1H,8-10 compared to cells treated with GST, but was not affected by treatment of cells with either FNIII1H,8-10ΔPHSRN or FNIII1H,8-10ΔRGD. These results indicate that both the synergy site and the RGD sequence are necessary for attenuation of PDGF-induced intracellular calcium release and provide evidence that α5β1 integrin ligation of FNIII9-10 is necessary for inhibitory activity.

### 3.4. Removal of β Strand a from FNIII8 Confers Functional Activity 

**Figure 5:**
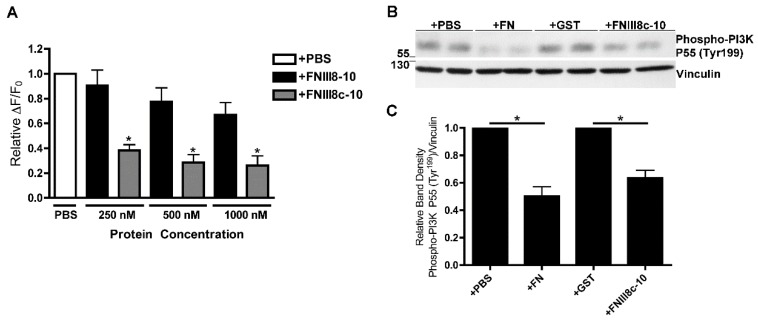
Effects of FNIII8c-10 on PDGF-induced calcium release and phosphoinositol 3-kinase (PI3K) activity. (**A**) FN-null MEFs were seeded on collagen-coated wells (**A**, 1.1 × 10^5^ cells/cm^2^; **B** and **C**, 6 × 10^4^ cells/cm^2^) and allowed to adhere for 4 h. (**A**) Cells were treated the indicated protein or equal volume of PBS and incubated for 20 h. Cells were loaded with Fluo-4, stimulated with 30 ng/mL PDGF, and monitored for changes in fluorescence intensity. Data are presented as relative change in fluorescence intensity from baseline (ΔF/F_0_) and are expressed as mean ± SEM of 3 experiments performed in quadruplicate. Values were normalized to the “+PBS” condition, which was set to 1. * Significantly different versus +PBS by ANOVA (*p* < 0.05). (**B**,**C**) Cells were treated with PBS, 200 nM FN, 1 μM FNIII8c-10 or 1 μM GST and incubated for 20 h. Cells were then treated with 30 ng/mL PDGF for 5 min. Cell lysates were analyzed by immunoblotting. (**B**) Representative immunoblot represents 1 of 3 independent experiments performed in duplicate. (**C**) Phospho-PI3K and vinculin band densities were quantified by densitometry. The ratio of the average net intensity of phospho-PI3K P55 (Tyr^199^) bands to the average net intensity of vinculin bands was determined. Values were normalized to the control “+PBS” and “+GST” conditions, which were set to 1. Data are presented as the average ratio ± SEM of 3 experiments performed in duplicate. * Significantly different by ANOVA (*p* < 0.05).

The cell-binding region of fibronectin is conformationally labile [32]. Moreover, molecular modeling studies predict that structural changes in FNIII modules adjacent to FNIII8-10 can induce conformational changes within the cell-binding domain [52]. FNIII1H and FNIIIEDBc were produced by removing the first 19 amino acids of FNIII1 and FNEDB respectively, corresponding to the removal of β-strand A from these modules [18,42]. Thus, we reasoned that removing β-strand A from FNIII8 (FNIII8c-10) might directly trigger changes in the conformation of FNIII9-10 to reveal the inhibitory activity. As shown in Figure 5A, pretreatment of cells with 250 nM FNIII8c-10 significantly attenuated PDGF-induced intracellular calcium release, whereas no inhibition was observed following pretreatment of cells with intact FNIII8-10 at concentrations up to 1 μM.

PDGF-induced intracellular calcium release is dependent upon activation of Class IA PI3K [53], which have either 55 kDa (P55) or 85 kDa (P85) regulatory subunits [54]. We showed previously that ECM fibronectin inhibits PDGF-induced phosphorylation of the P55 PI3K regulatory subunit [10]. To determine whether FNIII8c-10 similarly attenuates PDGF-induced PI3K activation, FN-null MEFs were pretreated with PBS, FN, GST, or FNIII8c-10 and then stimulated with PDGF. PDGF-induced phosphorylation of PI3K P55 (Tyr^199^) was significantly reduced in cells cultured in the presence of either fibronectin (Figure 5B,C; +PBS versus +FN) or FNIII8c-10 (Figure 5B,C; +GST versus +FNIII8c-10). Thus, removing β-strand A from FNIII8 is sufficient to reveal the inhibitory activity of FNIII9-10 in PDGF-PI3K-calcium signaling.

### 3.5. Molecular Modeling of FNIII8c-10

Molecular modeling of FNIII modules indicates that loss of hydrogen bonds between the A and B β-strands causes the opposing sheets of the β sandwich to rotate relative to one another and shift from a “twisted” to an “aligned” state [55,56]. As such, we hypothesized that removing the A β-strand from FNIII8 may introduce a similar conformational change that in turn, alters the relative positions of the two key-integrin-binding sites within FNIII9-10. Using the crystal structure of FNIII7-10 as reference, molecular dynamics (MD) simulations were performed for FNIII8-10 and FNIII8c-10 to compare the orientation and spacing of the RGD and synergy sites. Simulations were conducted using both united atom and all-atom approaches for FNIII8c-10, with good agreement obtained between final structural predictions. Given these results, dynamics simulations on FNIII8-10 used only the united atom model. 

**Figure 6:**
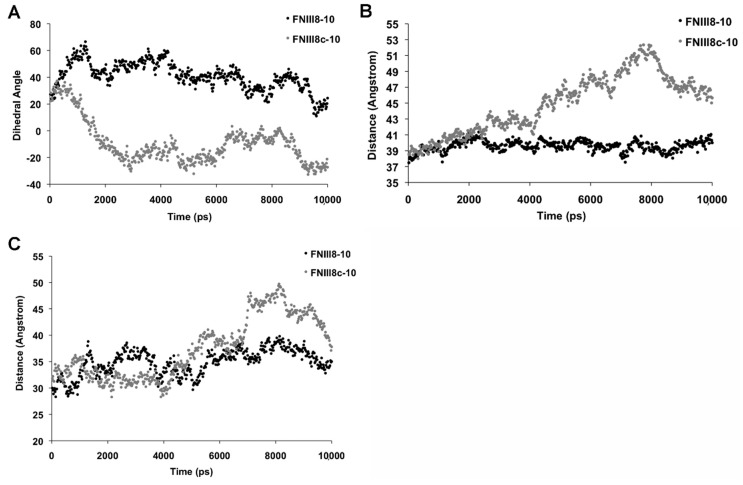
FNIII8-10 and FNIII8c-10 molecular dynamics (MD) output trajectories. Atomic coordinates were obtained through the Protein Data Bank (1FNF). Simulations were performed over a 10,000 ps simulation time. Shown are (**A**) dihedral rotational angle between the RGD and PHSRN synergy sites; (**B**) distance between the center of masses of FNIII9 and FNIII10; (**C**) distance between PHSRN and RGD sites.

**Figure 7:**
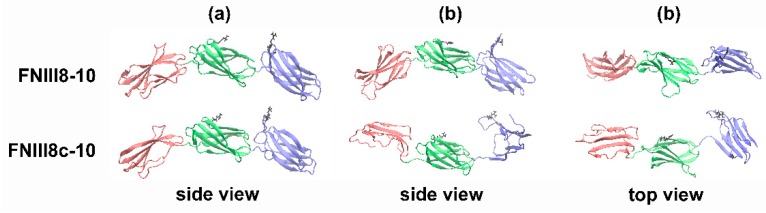
Ribbon diagrams of FNIII8-10 and FNIII8c-10 estimated by MD simulations. β-strands of FNIII8 or FNIII8c (red), FNIII9 (green) and FNIII10 (purple) are shown as ribbons, and R^1379^ and R^1493^ (black) are shown as ball and stick figures. Conformations of proteins are shown at the (**a**) beginning and (**b**) end of the 10000 ps simulations. Both side and top view images are shown for (b).

The dihedral angle between the RGD and PHSRN sites was measured over the course of the MD simulations. As shown in Figure 6A, the dihedral angle of the RGD and PHSRN sites of FNIII8-10 was 20.7° ± 0.9° (Figure 6A), similar to previous reports [14,57]. In contrast, well-defined rotational motions defined the FNIII8c-10 construct, as the PHSRN and RGD sites on FNIII8c-10 eclipsed each other over time and continued to rotate to an angle of −26.2° ± 0.9° (Figure 6A). FNIII8c-10 also displayed an extension of the overall protein structure, characterized by an increase in the distance between the center of the masses of FNIII9 and FNIII10 from 40.4 ± 0.1 Å in FNIII8-10 to 46.0 ± 0.2 Å in FNIII8c-10 (Figure 6B). Similarly, the distance between the RGD and PHSRN sites increased from 34.5 ± 0.2 Å in FNIII8-10 to 38.7 ± 0.3 Å in FNIII8c-10 (Figure 6C). The molecular structures of FNIII8-10 exhibited a high degree of similarity over the course of the simulations (Figure 6), indicating a relatively static structure. In contrast, MD simulations of FNIII8c-10 indicated that the truncated FNIII8 module partially unfolds, the distance between FNIII9 and FNIII10 increases, the modules rotate relative to one another, and the opposing β sheets of FNIII10 align parallel to one another. Ribbon diagrams of the conformations of FNIII8-10 and FNIII8c-10 at the beginning and end of the simulation period are shown in Figure 7.

**Figure 8:**
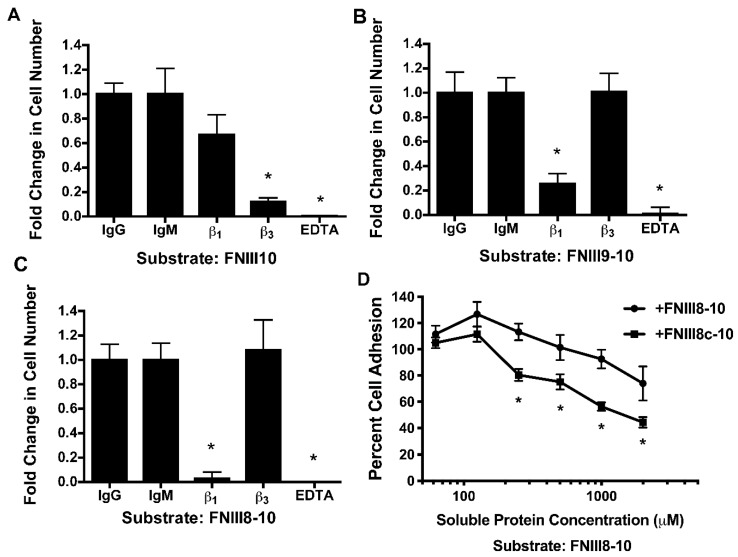
Cell attachment to fibronectin cell-binding domains. FN-null MEFs (9.4 × 10^4^ cells/cm^2^) were pre-incubated with (**A**–**C**) anti-β1 or anti-β3 integrin blocking antibodies, IgG or IgM controls, 10 mM EDTA, or (**D**) FNIII8-10 or FNIII8c-10 at indicated concentrations. Cells were seeded onto plates pre-coated with 125 nM FNIII10 (**A**), FNIII9-10 (**B**), or FNIII8-10 (**C**,**D**). Cell attachment was determined by staining with crystal violet, as described in the Methods section. (**A**–**C**) Data are presented as mean fold change over IgG or IgM controls of triplicate wells ± SEM of 3 independent experiments. * Significantly different from corresponding controls, *p* < 0.05 (ANOVA). (**D**) Data are presented as mean fold change over untreated cells (‘0′ nM) of triplicate wells ± SEM of 3 independent experiments. * Significantly different from corresponding concentration, *p* < 0.05 (*t*-test).

Cell attachment is a primary indicator of integrin-dependent binding and thus, cell adhesion assays were used to functionally compare FNIII8-10 and FNIII8c-10. As expected [32,35], cell adhesion to FNIII10 was blocked by anti-β3 integrin, but not by anti-β1 integrin antibodies (Figure 8A). In contrast, adhesion to FNIII9-10 or FNIII8-10 was blocked by anti-β1 integrin, but not by anti-β3 integrin antibodies (Figure 8B,C), indicating that cell adhesion to FNII9-10 and FNII8-10 is mediated by α5β1 integrins. Competition assays were next conducted using FNIII8-10 as the adhesive substrate in order to assess binding of FNIII8c-10 to α5β1 integrins in their native state. At concentrations of 125 μM and greater, soluble FNIII8c-10 was more effective at inhibiting cell attachment to FNIII8-10-coated plates than soluble FNIII8-10 (Figure 8D), indicating that FNIII8c-10 binds to α5β1 integrins and moreover, that the conformational changes induced by the FNIII8 truncation modestly increased integrin-binding affinity. 

## 4. Discussion

Understanding the mechanisms by which ECM fibronectin influences cell and tissue responses to PDGF is critical to designing targeted therapies for the wide range of pathologies characterized by aberrant PDGF signaling, including asthma, liver cirrhosis, atherosclerosis, pulmonary fibrosis, cancer, and chronic wounds. The ECM form of fibronectin selectively attenuates PDGF-induced PI3K activity and intracellular calcium release without affecting PDGF-induced activation of PDGF receptor β, protein kinase B (Akt), PLC-γ1, or extracellular signal-related kinase (ERK)-1/2 [10]. In the present study, we localized the bioactive effects of fibronectin to the α5β1 integrin-binding, FNIII9-10 domain. A novel, recombinant, cell-binding fibronectin fragment (FNIII8c-10) was developed and shown to attenuate PDGF-induced PI3K activation and intracellular calcium release. In contrast, intact FNIII8-10 fragments did not attenuate PDGF-induced intracellular calcium release, indicating that the removal of the A β-strand from FNIII8 is sufficient to confer the structure required to attenuate PDGF-induced intracellular calcium release. MD simulations indicated that the FNIII8c-10 fragment adopts a conformation in which the opposing β-sheets of III10 align, elongating the cell-binding domain by 6Å and permitting FNIII10 to rotate relative to FIII9. Additional studies provided evidence that the affinity of FNIII8c-10 for α5β1 integrins is increased modestly, relative to the intact FNIII8-10 fragment. Thus, this novel recombinant protein may show utility as a therapeutic that can selectively attenuate the PDGF-PI3K-calcium release cascade while leaving other PDGF pathways intact.

In contrast to several previous studies demonstrating a role for the matricryptic site of FNII1 in mediating the effects of ECM fibronectin [18,19,41,49,50], attenuation of PDGF-induced intracellular calcium release did not require the bioactive RWRPK sequence of FNIII1. Treatment of cell-assembled fibronectin matrices with the anti-FNIII1 blocking antibody, 9D2, did not reverse the effect of ECM fibronectin on PDGF-induced intracellular calcium release. Moreover, the FNIII1 mutant, FNIII1H,8-10ΔRWRK, was as effective at attenuating PDGF-induced intracellular calcium release as the non-mutant construct. As well, PDGF-induced intracellular calcium release was not attenuated by various FNIII1H-containing constructs that lacked integrin-binding sites (e.g., FNIII1H,2-4, FNIII1H,8-10ΔRGD and FNIII1H,8). Lastly, PDGF-induced intracellular calcium release was attenuated by FNIIIEDBc,8-10 to a similar extent as FNIII1H,8-10, confirming that FNIII1 is not required for inhibition of the PDGF-intracellular calcium release pathway. 

Changes to FNIII8-10 conformation have been observed in response to a variety of conditions, including force-induced unfolding of fibronectin [58], inclusion of FNEDB at the amino-terminus of FNIII8 [59], binding of gangliosides, glycosaminoglycans or gelatin to fibronectin [60], and binding of certain bacterial peptides to the amino-terminus of fibronectin [34]. Furthermore, structural perturbations initiated in one region of fibronectin can propagate intramolecularly to distant regions [34]. Data presented herein showed that placing a truncated FNIII fragment (FNIII1H, FNIIIEDBc or FNIII8c) N-terminal to the FNIII9-10 cell-binding modules shifted FNIII8-10 from an inactive to an active protein capable of inhibiting intracellular calcium release. In their native state, the two anti-parallel β-sheets of the FNIII modules are twisted with respect to one another by ~30° (i.e., “twisted state”) [52]. Our MD simulations indicated that in FNIII8c-10, the β strands adopt a more “aligned state”, in agreement with previous studies, showing that loss of hydrogen bonds between the A and B β-strands in FNIII modules shifts the conformation from the twisted to aligned state [55,56]. Our simulation results are also similar to those proposed during force-induced extension of fibronectin [52] and are consistent with the requirement that intact soluble fibronectin must undergo cell-mediated fibronectin fibril assembly in order to inhibit PDGF-induced calcium release [10]. 

Our results indicate that both the synergy site and the RGD sequence are necessary for attenuation of PDGF-induced intracellular calcium release. The crystal structure of FNIII7-10 shows that the RGD and PHSRN sites are separated by a distance of ~32 Å [27], while other studies indicate that ~37Å is the optimal distance for α5β1 integrin binding [61]. As reported previously [14], results from the present study indicate a distance of ~35 Å between the RGD and synergy sites in the intact construct, FNIII8-10. In FNIII8c-10, this distance increases to ~39 Å, which may contribute to the modest increase in affinity observed in the competition cell attachment studies. The FNIII8c-10 construct also exhibits increased flexibility in the link between FNIII9 and FNIII10, which may maximize interactions between the RGD sequence and α5β1 integrins. The ability of ECM fibronectin and FNIII8c-10 to inhibit intracellular calcium release, coupled with the inability of soluble fibronectin and FNIII8-10 to affect release, provide key evidence that an ECM-specific conformation of the integrin-binding domain of fibronectin regulates select responses to growth factor signaling. Moreover, results of our MD simulations provide insight into the structural reorganization that may occur within this region during fibronectin matrix assembly. Given the impact of growth factor signaling on cell function, it will be of interest to determine how the proposed conformational changes in FNIII8-10 manifest in fibronectin-α5β1 integrin interactions to, in turn, impact α5β1 integrin structure and signaling events. 

The increase in cytosolic calcium concentration evoked by PDGF activates downstream signaling by altering the conformation of intracellular signaling molecules. As one example, calcium is bound by calmodulin, which results in calmodulin undergoing a conformational change to adopt its active conformation [62]. In turn, activation of calmodulin allows calmodulin to bind to and activate various target proteins, such as calcium/calmodulin-dependent kinases (CaM-kinases), myosin light chain kinase and phosphorylase kinase [62]. In turn, CaM-kinases phosphorylate gene regulatory proteins to alter gene expression. The PDGF-induced calcium signaling cascade induces cell behaviors including differentiation [63], proliferation, migration [64], and changes in gene transcription [63]. In particular, PDGF-induced intracellular calcium release mediates increased transcription of messenger RNA for the ECM genes *collagen A1* and *fibronectin 1* [63]. PDGF-induced ECM gene transcription is blocked by inhibiting specific components of the PDGF-induced calcium release pathway, such as PLC-γ1 or the IP3 receptor [63].

The ECM form of fibronectin selectively attenuates PI3K activity and intracellular calcium release in response to PDGF [10]. In contrast, soluble fibronectin and fibronectin fragments have been shown to enhance PDGF-induced fibroblast and smooth muscle cell proliferation, migration, and PDGF-Rβ activation [65]. Fibronectin fragments can also accelerate growth factor-mediated wound closure and vascularization [65] and enhance PDGF-mediated cell survival [66]. The ability of fibronectin fragments to potentiate PDGF signaling are consistent with the results of the current study, which showed that intact cell-binding fibronectin fragments do not inhibit PDGF-induced calcium release. Rather, inhibition of calcium release was observed only in response to a conformationally-distinct fibronectin fragment. Together, these studies provide evidence that this inhibitory activity is cryptic in soluble fibronectin and exposed only after cell-mediated fibronectin fibril assembly. The ability of soluble and fibrillar fibronectins to differentially influence PDGF signaling highlights the versatility and usefulness of coupling protein structure to downstream activity. That is, dynamically coupling ECM structure to growth factor responsiveness may permit fine tuning of ECM synthesis and assembly and thus, tissue homeostasis.

The discovery that cell-assembled ECM fibronectin attenuates PDGF-induced PI3K activity and intracellular calcium release may be important in understanding the pathogenesis of conditions that are characterized by both altered cell responsiveness to PDGF and aberrant fibronectin matrix assembly. As one example, fibrosis is characterized by substantial remodeling of the ECM, excess proliferation of myofibroblasts that are abnormally responsive to PDGF, and enhanced deposition of ECM proteins [67]. Together, results of the current and previous [10] studies suggest that ECM fibronectin-mediated attenuation of PDGF-induced intracellular calcium release is dependent upon a unique conformation of the cell-binding region of fibronectin, and that this structural configuration is not expressed either by soluble fibronectin or proteolytic fibronectin fragments released through interdomain cleavage. PDGF-induced intracellular calcium release mediates PDGF-induced fibroblast proliferation and ECM gene expression [1,63,64]. These observations imply that a negative feedback loop, by which cell-assembled ECM fibronectin attenuates PDGF-induced ECM gene expression, would be sensitive to a specific conformation of the cell-binding domain of ECM fibronectin fibrils, and by extension, a specific conformation of α5β1 integrins. We speculate that abnormal ECM remodeling during fibrosis may reduce the overall expression of this inhibitory conformation of ECM fibronectin and thus, relieve the putative negative feedback loop to, in turn, sustain matrix protein production. Fibrotic diseases such as pulmonary fibrosis, sclerosis, liver cirrhosis, and cardiovascular diseases contribute to over 45% of deaths in the developed world [68]. As such, FNIII8c-10 may be an attractive candidate for therapeutic development as a way to selectively attenuate PDGF-induced ECM protein production in pathologies characterized by both enhanced PDGF signaling and excess ECM protein deposition.

## 5. Conclusions

A novel, recombinant, cell-binding fragment of fibronectin was shown to inhibit both PI3K activation and intracellular calcium release in response to PDGF. This fragment was produced by removing β-strand A from the amino-terminus of FNIII8. Molecular dynamics simulations of FNIII8c-10 indicated that as a result of the FNIII8 truncation, FNIII9 and FNIII10 adopt an aligned, extended conformation with a unique rotational angle between the RGD and PHSRN sites. PDGF-induced intracellular calcium release was attenuated by soluble fibronectin fragments that bind to α5β1, and not αvβ3, integrins. Previous studies [10] demonstrated that the ECM form of fibronectin inhibits PDGF-induced intracellular calcium release. Taken together, these results indicate that an aligned, ECM-specific conformation of FNIII9-10 is required to attenuate PDGF-induced intracellular calcium release.

## References

[1] Heldin C.H., Westermark B. (1999). Mechanism of action and in vivo role of platelet-derived growth factor. Physiol. Rev..

[2] Yao R., Cooper G.M. (1995). Requirement for phosphatidylinositol-3 kinase in the prevention of apoptosis by nerve growth factor. Science.

[3] Siegbahn A., Hammacher A., Westermark B., Heldin C.H. (1990). Differential effects of the various isoforms of platelet-derived growth factor on chemotaxis of fibroblasts, monocytes, and granulocytes. J. Clin. Investig..

[4] Clark R.A., Folkvord J.M., Hart C.E., Murray M.J., McPherson J.M. (1989). Platelet isoforms of platelet-derived growth factor stimulate fibroblasts to contract collagen matrices. J. Clin. Investig..

[5] Gullberg D., Tingstrom A., Thuresson A.C., Olsson L., Terracio L., Borg T.K., Rubin K. (1990). Beta 1 integrin-mediated collagen gel contraction is stimulated by PDGF. Exp. Cell Res..

[6] Falanga V. (2005). Wound healing and its impairment in the diabetic foot. Lancet.

[7] Nagai M.K., Embil J.M. (2002). Becaplermin: Recombinant platelet derived growth factor, a new treatment for healing diabetic foot ulcers. Expert Opin. Biol..

[8] Nakamura K., Matsubara H., Akagi S., Sarashina T., Ejiri K., Kawakita N., Yoshida M., Miyoshi T., Watanabe A., Nishii N. (2017). Nanoparticle-Mediated Drug Delivery System for Pulmonary Arterial Hypertension. J. Clin. Med..

[9] Wollin L., Wex E., Pautsch A., Schnapp G., Hostettler K.E., Stowasser S., Kolb M. (2015). Mode of action of nintedanib in the treatment of idiopathic pulmonary fibrosis. Eur. Respir. J..

[10] Farrar C.S., Hocking D.C. (2018). Assembly of fibronectin fibrils selectively attenuates platelet-derived growth factor-induced intracellular calcium release in fibroblasts. J. Biol. Chem..

[11] Singh P., Carraher C., Schwarzbauer J.E. (2010). Assembly of fibronectin extracellular matrix. Annu. Rev. Cell Dev. Biol..

[12] Baneyx G., Baugh L., Vogel V. (2002). Fibronectin extension and unfolding within cell matrix fibrils controlled by cytoskeletal tension. Proc. Natl. Acad. Sci. USA.

[13] Brown A.C., Dysart M.M., Clarke K.C., Stabenfeldt S.E., Barker T.H. (2015). Integrin alpha3beta1 Binding to Fibronectin Is Dependent on the Ninth Type III Repeat. J. Biol. Chem..

[14] Krammer A., Craig D., Thomas W.E., Schulten K., Vogel V. (2002). A structural model for force regulated integrin binding to fibronectin’s RGD-synergy site. Matrix Biol..

[15] Zhong C., Chrzanowska-Wodnicka M., Brown J., Shaub A., Belkin A.M., Burridge K. (1998). Rho-mediated contractility exposes a cryptic site in fibronectin and induces fibronectin matrix assembly. J. Cell Biol..

[16] Cao L., Zeller M.K., Fiore V.F., Strane P., Bermudez H., Barker T.H. (2012). Phage-based molecular probes that discriminate force-induced structural states of fibronectin in vivo. Proc. Natl. Acad. Sci. USA.

[17] Ohashi T., Lemmon C.A., Erickson H.P. (2017). Fibronectin Conformation and Assembly: Analysis of Fibronectin Deletion Mutants and Fibronectin Glomerulopathy (GFND) Mutants. Biochemistry.

[18] Hocking D.C., Kowalski K. (2002). A cryptic fragment from fibronectin’s III1 module localizes to lipid rafts and stimulates cell growth and contractility. J. Cell Biol..

[19] Hocking D.C., Titus P.A., Sumagin R., Sarelius I.H. (2008). Extracellular matrix fibronectin mechanically couples skeletal muscle contraction with local vasodilation. Circ. Res..

[20] Pierschbacher M.D., Ruoslahti E. (1984). Cell attachment activity of fibronectin can be duplicated by small synthetic fragments of the molecule. Nature.

[21] Ruoslahti E., Pierschbacher M.D. (1987). New perspectives in cell adhesion: RGD and integrins. Science.

[22] Altroff H., van der Walle C.F., Asselin J., Fairless R., Campbell I.D., Mardon H.J. (2001). The eighth FIII domain of human fibronectin promotes integrin alpha5beta1 binding via stabilization of the ninth FIII domain. J. Biol. Chem..

[23] Chen W., Culp L.A. (1996). Adhesion mediated by fibronectin’s alternatively spliced EDb (EIIIB) and its neighboring type III repeats. Exp. Cell Res..

[24] Wong J.Y., Weng Z., Moll S., Kim S., Brown C.T. (2002). Identification and validation of a novel cell-recognition site (KNEED) on the 8th type III domain of fibronectin. Biomaterials.

[25] Aota S., Nomizu M., Yamada K.M. (1994). The short amino acid sequence Pro-His-Ser-Arg-Asn in human fibronectin enhances cell-adhesive function. J. Biol. Chem..

[26] Redick S.D., Settles D.L., Briscoe G., Erickson H.P. (2000). Defining fibronectin’s cell adhesion synergy site by site-directed mutagenesis. J. Cell Biol..

[27] Leahy D.J., Aukhil I., Erickson H.P. (1996). 2.0 A crystal structure of a four-domain segment of human fibronectin encompassing the RGD loop and synergy region. Cell.

[28] Petrie T.A., Capadona J.R., Reyes C.D., Garcia A.J. (2006). Integrin specificity and enhanced cellular activities associated with surfaces presenting a recombinant fibronectin fragment compared to RGD supports. Biomaterials.

[29] Altroff H., Schlinkert R., van der Walle C.F., Bernini A., Campbell I.D., Werner J.M., Mardon H.J. (2004). Interdomain tilt angle determines integrin-dependent function of the ninth and tenth FIII domains of human fibronectin. J. Biol. Chem..

[30] Martino M.M., Mochizuki M., Rothenfluh D.A., Rempel S.A., Hubbell J.A., Barker T.H. (2009). Controlling integrin specificity and stem cell differentiation in 2D and 3D environments through regulation of fibronectin domain stability. Biomaterials.

[31] Ambesi A., McKeown-Longo P.J. (2014). Conformational remodeling of the fibronectin matrix selectively regulates VEGF signaling. J. Cell Sci..

[32] Cao L., Nicosia J., Larouche J., Zhang Y., Bachman H., Brown A.C., Holmgren L., Barker T.H. (2017). Detection of an Integrin-Binding Mechanoswitch within Fibronectin during Tissue Formation and Fibrosis. ACS Nano.

[33] Wang K., Andresen Eguiluz R.C., Wu F., Seo B.R., Fischbach C., Gourdon D. (2015). Stiffening and unfolding of early deposited-fibronectin increase proangiogenic factor secretion by breast cancer-associated stromal cells. Biomaterials.

[34] Liang X., Garcia B.L., Visai L., Prabhakaran S., Meenan N.A., Potts J.R., Humphries M.J., Hook M. (2016). Allosteric Regulation of Fibronectin/alpha5beta1 Interaction by Fibronectin-Binding MSCRAMMs. PLoS ONE.

[35] Roy D.C., Hocking D.C. (2013). Recombinant fibronectin matrix mimetics specify integrin adhesion and extracellular matrix assembly. Tissue Eng. Part. A.

[36] Akiyama S.K., Yamada S.S., Chen W.T., Yamada K.M. (1989). Analysis of fibronectin receptor function with monoclonal antibodies: Roles in cell adhesion, migration, matrix assembly, and cytoskeletal organization. J. Cell Biol..

[37] Chernousov M.A., Fogerty F.J., Koteliansky V.E., Mosher D.F. (1991). Role of the I-9 and III-1 modules of fibronectin in formation of an extracellular fibronectin matrix. J. Biol. Chem..

[38] Sottile J., Hocking D.C., Swiatek P.J. (1998). Fibronectin matrix assembly enhances adhesion-dependent cell growth. J. Cell Sci..

[39] Hocking D.C., Smith R.K., McKeown-Longo P.J. (1996). A novel role for the integrin-binding III-10 module in fibronectin matrix assembly. J. Cell Biol..

[40] Hocking D.C., Sottile J., Reho T., Fassler R., McKeown-Longo P.J. (1999). Inhibition of fibronectin matrix assembly by the heparin-binding domain of vitronectin. J. Biol. Chem..

[41] Gui L., Wojciechowski K., Gildner C.D., Nedelkovska H., Hocking D.C. (2006). Identification of the heparin-binding determinants within fibronectin repeat III1: Role in cell spreading and growth. J. Biol. Chem..

[42] Roy D.C., Wilke-Mounts S.J., Hocking D.C. (2011). Chimeric fibronectin matrix mimetic as a functional growth- and migration-promoting adhesive substrate. Biomaterials.

[43] Brennan J.R., Hocking D.C. (2016). Cooperative effects of fibronectin matrix assembly and initial cell-substrate adhesion strength in cellular self-assembly. Acta Biomater..

[44] Hocking D.C., Sottile J., McKeown-Longo P.J. (1994). Fibronectin’s III-1 module contains a conformation-dependent binding site for the amino-terminal region of fibronectin. J. Biol. Chem..

[45] Straub S.V., Giovannucci D.R., Yule D.I. (2000). Calcium wave propagation in pancreatic acinar cells: Functional interaction of inositol 1,4,5-trisphosphate receptors, ryanodine receptors, and mitochondria. J. Gen. Physiol..

[46] Johenning F.W., Zochowski M., Conway S.J., Holmes A.B., Koulen P., Ehrlich B.E. (2002). Distinct intracellular calcium transients in neurites and somata integrate neuronal signals. J. Neurosci..

[47] Qui D., Shenkin P.S., Hollinger F.P., Still W.C. (1997). The GB/SA continuum model for solvation. A fast analytical method for the calculation of approximate born radii. J. Phys. Chem. A.

[48] Moolenaar W.H., Tertoolen L.G., de Laat S.W. (1984). Growth factors immediately raise cytoplasmic free Ca2+ in human fibroblasts. J. Biol. Chem..

[49] Sarelius I.H., Titus P.A., Maimon N., Okech W., Wilke-Mounts S.J., Brennan J.R., Hocking D.C. (2016). Extracellular matrix fibronectin initiates endothelium-dependent arteriolar dilatation via the heparin-binding, matricryptic RWRPK sequence of the first type III repeat of fibrillar fibronectin. J. Physiol..

[50] Okech W., Abberton K.M., Kuebel J.M., Hocking D.C., Sarelius I.H. (2016). Extracellular matrix fibronectin mediates an endothelial cell response to shear stress via the heparin-binding, matricryptic RWRPK sequence of FNIII1H. Am. J. Physiol. Heart Circ. Physiol..

[51] Garcia A.J., Schwarzbauer J.E., Boettiger D. (2002). Distinct activation states of alpha5beta1 integrin show differential binding to RGD and synergy domains of fibronectin. Biochemistry.

[52] Craig D., Krammer A., Schulten K., Vogel V. (2001). Comparison of the early stages of forced unfolding for fibronectin type III modules. Proc. Natl. Acad. Sci. USA.

[53] Heldin C.H., Ostman A., Ronnstrand L. (1998). Signal transduction via platelet-derived growth factor receptors. Biochim. Biophys. Acta.

[54] Vanhaesebroeck B., Guillermet-Guibert J., Graupera M., Bilanges B. (2010). The emerging mechanisms of isoform-specific PI3K signalling. Nat. Rev. Mol. Cell Biol..

[55] Gao M., Craig D., Lequin O., Campbell I.D., Vogel V., Schulten K. (2003). Structure and functional significance of mechanically unfolded fibronectin type III1 intermediates. Proc. Natl. Acad. Sci. USA.

[56] Gao M., Craig D., Vogel V., Schulten K. (2002). Identifying unfolding intermediates of FN-III(10) by steered molecular dynamics. J. Mol. Biol..

[57] Pan D., Song Y. (2001). Role of altered sialylation of the I-like domain of beta1 integrin in the binding of fibronectin to beta1 integrin: Thermodynamics and conformational analyses. Biophys. J..

[58] Smith M.L., Gourdon D., Little W.C., Kubow K.E., Eguiluz R.A., Luna-Morris S., Vogel V. (2007). Force-induced unfolding of fibronectin in the extracellular matrix of living cells. PLoS Biol..

[59] Bencharit S., Cui C.B., Siddiqui A., Howard-Williams E.L., Sondek J., Zuobi-Hasona K., Aukhil I. (2007). Structural insights into fibronectin type III domain-mediated signaling. J. Mol. Biol..

[60] Ugarova T.P., Zamarron C., Veklich Y., Bowditch R.D., Ginsberg M.H., Weisel J.W., Plow E.F. (1995). Conformational transitions in the cell binding domain of fibronectin. Biochemistry.

[61] Craig J.A., Rexeisen E.L., Mardilovich A., Shroff K., Kokkoli E. (2008). Effect of linker and spacer on the design of a fibronectin-mimetic peptide evaluated via cell studies and AFM adhesion forces. Langmuir.

[62] Clapham D.E. (2007). Calcium signaling. Cell.

[63] Mukherjee S., Duan F., Kolb M.R., Janssen L.J. (2013). Platelet derived growth factor-evoked Ca2+ wave and matrix gene expression through phospholipase C in human pulmonary fibroblast. Int. J. Biochem. Cell Biol..

[64] Hollenbeck S.T., Nelson P.R., Yamamura S., Faries P.L., Liu B., Kent K.C. (2004). Intracellular calcium transients are necessary for platelet-derived growth factor but not extracellular matrix protein-induced vascular smooth muscle cell migration. J. Vasc. Surg..

[65] Martino M.M., Tortelli F., Mochizuki M., Traub S., Ben-David D., Kuhn G.A., Muller R., Livne E., Eming S.A., Hubbell J.A. (2011). Engineering the growth factor microenvironment with fibronectin domains to promote wound and bone tissue healing. Sci. Transl. Med..

[66] Lin F., Zhu J., Tonnesen M.G., Taira B.R., McClain S.A., Singer A.J., Clark R.A. (2014). Fibronectin peptides that bind PDGF-BB enhance survival of cells and tissue under stress. J. Invest. Derm..

[67] Wynn T.A. (2007). Common and unique mechanisms regulate fibrosis in various fibroproliferative diseases. J. Clin. Investig..

[68] Cox T.R., Erler J.T. (2011). Remodeling and homeostasis of the extracellular matrix: Implications for fibrotic diseases and cancer. Dis. Model. Mech..

